# The clinical application value of phase angle of six parts in nutritional evaluation of tumor patients

**DOI:** 10.1007/s00520-022-07240-x

**Published:** 2022-06-27

**Authors:** Xiaoling Zhang, Jialei Zhang, Yunyi Du, Xiaoyu Wu, Yali Chang, Weiling Li, Yaqin Liu, Wenqing Hu, Jun Zhao

**Affiliations:** 1 Department of Oncology, Changzhi People’s Hospital Affiliated to Changzhi Medical College, Changzhi, 046000 Shanxi Province China; 2 Department of Anesthesiology, Changzhi People’s Hospital Affiliated to Changzhi Medical College, Changzhi, 046000 Shanxi Province China; 3 Department of Respiration, Changzhi Medical College, Changzhi, 046000 Shanxi China; 4 Gastrointestinal Surgery Department, Changzhi People’s Hospital Affiliated to Changzhi Medical College, Changzhi, 046000 Shanxi Province China

**Keywords:** Phase angle, Six parts, Nutritional assessment, Prediction model, PG-SGA

## Abstract

**Objectives:**

The purpose of this study was to explore the clinical application value of phase angle (PA) of six parts in the nutritional evaluation and construct a prediction model for diagnosing malnutrition of tumor patients.

**Methods:**

A total of 1129 patients with malignant tumors were analyzed retrospectively. The age, sex, tumor location and body mass index (BMI) of the patients were collected, and PA of six parts was measured. The Patient Subjective Global Assessment (PG-SGA) was used to evaluate the nutritional status of each patient.

**Results:**

According to the PG-SGA, 66.5% (*n* = 750) of the patients were evaluated as malnourished. Patients under the age of 65 had higher PA values. The PA value of men was higher than that of women (except PA-RL). In different disease groups, the PA-RA and PA-TR values were significantly different. In our study, PA value increases with BMI and decreases with PG-SGA (except PG-SGA 0–1 group). Multivariate regression analysis indicates that the age (HR = 1.051, 95% CI 1.037–1.066, *P* < 0.001), BMI (HR = 0.885, 95% CI 0.849–0.924, *P* < 0.001), and PA-WB (HR = 0.615, 95% CI 0.546–0.692, *P* < 0.001) were independent significant predictors associated with malnutrition. The AUC of the prediction model is 0.7631 (*p* < 0.001), indicating that the model including age, BMI, and PA-WB has certain diagnostic value for the diagnosis of malnutrition.

**Conclusion:**

The PA-WB is an independent prognostic factor of malnutrition. The prediction model constructed by age, BMI, and PA-WB can be used as a useful tool for nutritional evaluation of tumor patients.

**Trial registration:**

Clinical Trial No.: ChiCTR2100047858.

## Introduction

Cancer patients are at high risk of malnutrition. Nutritional status is closely related to patient quality of life, tumor-related treatment results, and prognosis [4, 6]. Therefore, active and effective early intervention are of great significance for patients with malignant tumors. The first step in nutritional standardized treatment is nutritional assessment. BMI is an index that is commonly used internationally to evaluate human nutritional status, obesity, and health [5, 14]. The PG-SGA was developed on the basis of the subjective global assessment (SGA) that was first proposed by Ottery FD in 1994 [11]. The PG-SGA is an effective and specific nutritional evaluation tool specifically designed for cancer patients. It has been recommended and widely used by the American Dietetic Association (ADA) and the Cancer Nutrition and Supportive Treatment Committee of the Chinese Anti-Cancer Association (CSONSC) [1, 7, 8, 12, 13, 15, 16].

PA is considered as a good tool to assess the intracellular compartment, cell integrity, and nutritional status of cancer patients, which is derived from bioelectrical impedance analysis (BIA) [3]. This study aims to explore the clinical application value of PA in the nutritional assessment of tumor patients.

## Methods

### Case selection and general information

A retrospective analysis was performed with inpatients in the tumor center of our hospital from June 2020 to February 2021. All patients included in this study were diagnosed with malignant tumors and had able-bodied limbs. The age, sex, tumor location, and body mass index (BMI) of the patients were collected. Six parts of PA were assessed within 24 h of the patient’s hospitalization, and the following were measured separately: PA of left arm (PA-LA), PA of right arm (PA-RA), PA of left leg (PA-LL), PA of right leg (PA-RL), PA of trunk (PA-TR), and PA of whole body (PA-WB). The PG-SGA was used to evaluate the nutritional status of each patient. A total of 1176 patients were eligible, of whom 47 patients were excluded due to lack of PA data. Therefore, 1129 patients were included in this study.

### Quality control of BMI, PG-SGA, and PA

BMI and PA were evaluated by the Hikang H-KEY350 body composition analyzer produced by Beijing Sihai Huachen Technology Co., LTD. The test requirements were as follows: (1) strong exercise or other physical activity is not recommended within 2 h before the test; (2) it is not recommended to eat within 2 h before the test; (3) it is not recommended to bathe within 2 h before the test; (4) the indoor temperature is kept between 21 and 25℃ better; (5) it is recommended to go to the bathroom before the test, because human excrement will temporarily change body composition.

The PG-SGA operation DVD recorded by the American College of Nutrition and Dietetics (AND) was used for PG-SGA evaluation.

### Grouping of BMI and PG-SGA

BMI is the number obtained by dividing the weight (unit: kg) by the square of the height (unit: m), and BMI is often used for clinical nutritional diagnosis. According to China’s standards, BMI is divided into 4 groups: underweight, BMI < 18.5 kg/m^2^; normal weight, 18.5 ≤ BMI < 24.0 kg/m^2^; overweight, 24.0 ≤ BMI < 28.0 kg/m^2^; and obesity group, BMI ≥ 28.0 kg/m^2^.

PG-SGA was divided into four groups according to the scores recommended by the Cancer Nutrition and Supportive Treatment Professional Committee of the Chinese Anti-Cancer Association: the well-nourished group (0–1 points), the suspected malnutrition group (2–3 points), the moderate malnutrition group (4–8 points), and the severe malnutrition group (≥ 9 points).

### Statistical methods

Statistical analysis was performed using IBM SPSS Statistics 26.0 which is produced by SPSS Inc and figures were made using GraphPad Prism 8.3.0. The median (lower quartile, upper quartile) was used to describe the PA values of the six parts. The statistical analysis used two-sided hypothesis test, *p* < 0.05, which was statistically significant. The Kolmogorov–Smirnov (K-S) normality test was performed on the data. For sex and age, the Mann–Whitney *U* test was used to analyze differences. For the BMI, PG-SGA, and different disease groups, the Kruskal–Wallis *H* test was used for statistical analysis. The correlations between six parts of PA were assessed by Spearman.

Whether the patient was malnourished was taken as the dependent variable, and the relevant factors involved in the study (age, gender, disease location, BMI, PA) were taken as the independent variables. Univariate analysis was performed (*t*-test, rank sum test, or chi square test), and then the independent variables with statistical significance (or close to statistical significance) by univariate analysis and clinically significant variables were subjected to multivariate logistic regression. Receiver operating characteristic (ROC) analysis was performed to evaluate the accuracy of model prediction.

## Results

### Characteristics of 1129 patients

The characteristics of the patients are summarized in Table 1. In this sample of hospitalized patients, the median (range) age was 60.1 (31–86) years. There were more patients younger than 65 years old (64.3%, *n* = 726) than those older than 65 years old(35.7%, *n* = 403), and more male (54.1%, *n* = 611) than female (45.9%, *n* = 518). The most frequent types of tumors were COADREAD (23.8%, *n* = 269) and STAD/EGJC (23.8%, *n* = 269) followed by NSCLC (11.5%, *n* = 130), GYNE (11.1%, *n* = 125), and ESCA (11.1%, *n* = 125), which represents a total of 81.3% (*n* = 918) of the sample. According to the BMI, 14.6% (*n* = 165) of the patients were severely malnourished (BMI < 18.5 kg/m^2^). The BMI of most patients ranged from 18.5 to 24.0, accounting for 54.9% (*n* = 620). According to the PG-SGA, 66.5% (*n* = 750) of the patients were moderately or severely malnourished (PG-SGA ≥ 4). The number of cases with PG-SGA score of 0–1 is very small (1.8%, *n* = 20).

**Table 1:** Characteristics of 1129 patients

Variables		*N*	%	Median (range)
Age	< 65	726	64.3	60.10 (31 ~ 86)
	≥ 65	403	35.7	
Sex	MALE	611	54.1	
	FEMALE	518	45.9	
Disease	NSCLC	130	11.5	
	GYNE	125	11.1	
	COAD/READ	269	23.8	
	BRCA	63	5.6	
	ESCA	125	11.1	
	STAD/EGJC	269	23.8	
	PAAD	26	2.3	
	Others	122	10.8	
BMI	< 18.5	165	14.6	22.52(15.9 ~ 30.9)
	18.5 ~ 24.0	620	54.9	
	24.0 ~ 28.0	270	23.9	
	≥ 28.0	74	6.6	
PG-SGA	0–1	20	1.8	5.96(1 ~ 24)
	2–3	359	31.8	
	4–8	528	46.8	
	≥ 9	222	19.7	

Abbreviations: *NSCLC*, non-small-cell carcinoma; *GYNE*, gynecological malignancies; *COAD/READ*, colon adenocarcinoma/rectum adenocarcinoma; *BRCA*, breast invasive carcinoma; *ESCA*, esophageal carcinoma; *STAD/EGJC*, stomach adenocarcinoma/esophagogastric junction cancer; *PAAD*, pancreatic adenocarcinoma; *Others*, other malignancies

### Pairwise comparison and correlation analysis of PA in six parts of patients

**Fig. 1:**
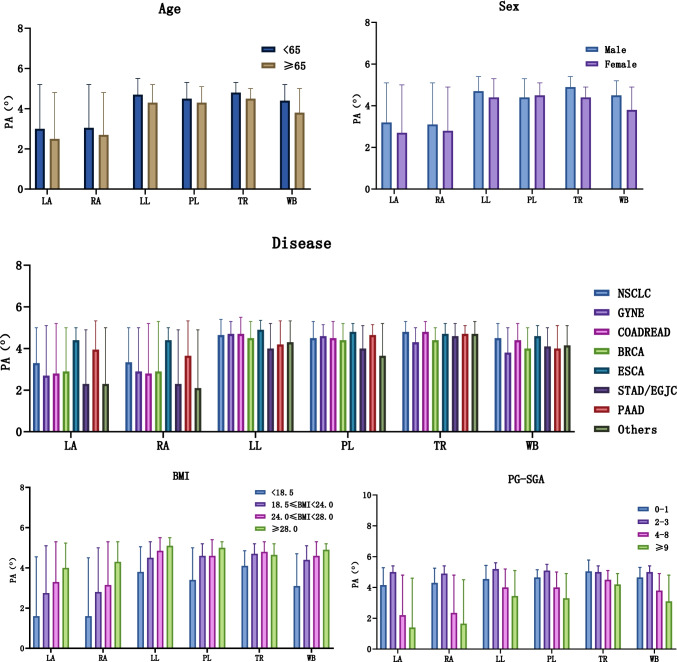
Stratified analysis of PA in six parts of tumor patients

The PA of six parts was analyzed from the perspectives of different age, sex, disease, BMI, and PG-SGA grade (Fig. 1). At the age of 65 years, there were significant differences in PA of six parts of the body, and individuals under 65 years had higher PA. The PA-LA, PA-RA, PA-LL, PA-TR, and PA-WB differed significantly according to sex, with higher values in men than in women. There was no difference in PA-RL by sex. In different disease groups, the PA-RA and PA-TR values were significantly different, but there was no significant difference in other parameters. The PA values of the six body parts were notably positively correlated with BMI group. The larger the BMI value, the larger the PA value. The PA value was negatively correlated with PG-SGA (2–3), (4–8), (≥ 9), and the PG-SGA (0–1) group was not significantly correlated with the PA value.

**Fig. 2:**
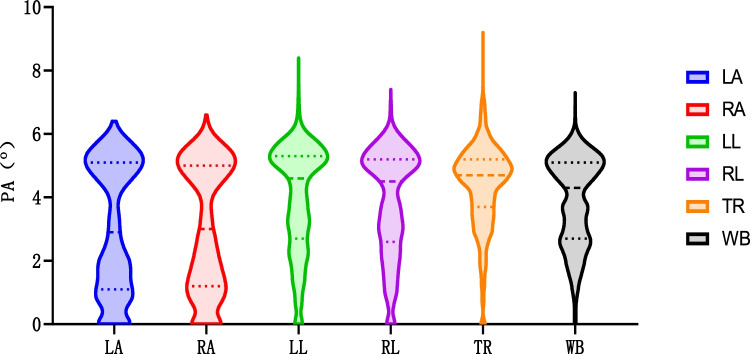
Violin diagram of PA in six parts of tumor patients

**Fig. 3:**
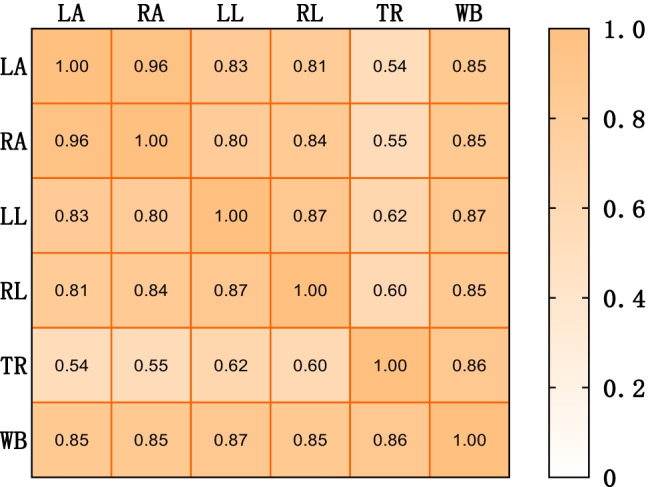
Correlation analysis of PA in six parts of tumor patients

In order to intuitively show the concentration and dispersion of the PA of the six parts, we draw the violin diagram (Fig. 2) and show the median and quartile on the diagram. From the figure, we can intuitively see that among the six parts, PA-TR has the best data concentration, followed by PA-LL, PA-RL, and PA-WB. From the correlation analysis heat map of six parts (Fig. 3), the overall correlation between PA-WB and the other five parts was the best, and the correlation between PA-TR and other parts was poor. Therefore, in the subsequent regression analysis, we intend to use the PA-WB data with relatively good data concentration and the best overall correlation with other parts for analysis.

### Logistic regression

Table 2 provides the results of the univariate and multivariate logistic regression analysis for malnutrition, including the variables age, sex, disease, BMI, and PA-WB. We regard 4 as PG-SGA cut-off point and PG-SGA ≥ 4 as malnutrition. We found that sex had no statistical significance with malnutrition, *p* = 0.85, while other variables had statistical significance (*p* < 0.001). However, according to the results of the “Pairwise comparison and correlation analysis of PA in six parts of patients” section, sex has an impact on PA value. Therefore, from clinical considerations, sex is still included in multivariate analysis. Finally, age, sex, disease, BMI, and PA-WB were included to multivariate analysis. The multivariate regression analysis indicates that the older patients had the higher risk of malnutrition (HR = 1.051, 95% CI 1.037–1.066, *p* < 0.001). The increase of BMI could reduce the risk of malnutrition (HR = 0.885, 95% CI 0.849–0.924, *p* < 0.001). The higher PA-WB, the lower the risk of malnutrition (HR = 0.615, 95% CI 0.546–0.692, *p* < 0.001). The results showed that age, BMI, and PA-WB were independent significant predictors associated with malnutrition.

**Table 2:** Univariate and multivariate analysis of malnutrition in tumor patients

Clinical features	Univariate analysis			Multivariate analysis		
	*B*	HR (95%CI)	*p* value	*B*	Corrected HR (95%CI)	*p* value
Age	0.052	1.054(1.039–1.068)	0.000	0.050	1.051(1.037–1.066)	0.000
Sex	0.196	1.217(0.894–1.658)	0.850	0.134	1.143(0.853–1.532)	0.370
Disease	0.039	1.040(0.973–1.112)	0.000	0.028	1.028(0.963–1.099)	0.408
BMI	− 0.120	0.887(0.850–0.925)	0.000	− 0.122	0.885(0.849–0.924)	0.000
PA-WB	− 0.062	0.940(0.435–2.033)	0.000	− 0.486	0.615(0.546–0.692)	0.000

*B*, regression coefficient; *HR*, hazard ratio

### Model evaluation

**Fig. 4:**
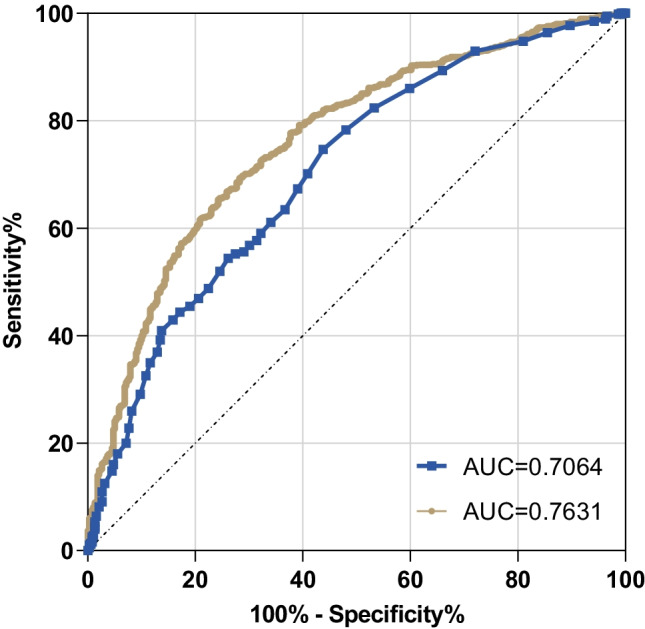
ROC for model validation

In summary, age, BMI, and PA-WB were included to construct a prediction model of malnutrition. In order to evaluate the malnutrition diagnosis ability of PA-WB and prediction model, we drew the ROC curve (Fig. 4). We can see that the ROC curve of the prediction model was mostly at the upper left of PA-WB, indicating that the diagnostic value of the prediction model is mightily higher than that of PA-WB. Table 3 shows that the AUC (area under the ROC) of malnutrition predicted by PA-WB was 0.7064, which is statistically significant compared with 0.5 (*p* < 0.001). The AUC of PA-WB combined with age and BMI in the diagnosis of malnutrition was 0.7631, which was statistically significant compared with 0.5 (*p* < 0.001).

**Table 3:** AUC of PA-WB and prediction model

Variable	Area	SE^a^	Asymptotic sig.^b^	Asymptotic 95% confidence interval	
				Lower bound	Upper bound
PA-WB	0.7064	0.0163	0.000	0.6744	0.7384
Prediction model	0.7631	0.0149	0.000	0.7339	0.7922

^a^Under the nonparametric assumption

^b^Null hypothesis: true area = 0.5

The statistic *Z* was calculated according to the AUC and SE of the two ROC curves. The formula is substituted to calculate *Z* = $\frac{\left|\mathrm{AUC}1-\mathrm{AUC}2\right|}{\sqrt{{\mathrm{SE}1}^{2}+{\mathrm{SE}2}^{2}}}$=$\frac{\left|0.7631-0.7064\right|}{ \sqrt{{0.0149}^{2}+{0.0163}^{2}}}=$ 2.577, *p* = (1-NORMSDIST (Z value))$\times$ 2 = (1-NORMSDIST (2.577))$\times$ 2 = (1–0.996)$\times$ 2 = 0.008 < 0.05. The difference of AUC was statistically significant(*p* < 0.05).

## Discussion

The first step of standardized cancer nutritional therapy is nutritional assessment. The purpose of the study was to explore the clinical application value of PA of six parts in the nutritional evaluation and construct a prediction model for diagnosing malnutrition of tumor patients. Our study analyzed 1129 patients with malignant tumors and found that PA can be used in nutritional assessment.

In our study, we found that PA in six parts of the body varied according to age, sex, and disease. In addition, we found that the PA value of the trunk is very concentrated, but its correlation with other parts is poor, while the PA-WB data is relatively concentrated and has the best overall correlation with other parts, which may be the key observation of our follow-up study. We found that PA values were higher in people under the age of 65. Except PA-RL, the PA values of other parts in men were higher than those in women. Therefore, in nutritional evaluation, we should pay attention to the differences of PA values with age and sex, and there are some differences in PA values of different body parts.

The World Health Organization (WHO) classifies people with a BMI in the range of 18.5–24.9 kg/m^2^ as normal weight. However, due to the differences in physiques between individuals from eastern and western countries, China has introduced another reference standard, setting the BMI index between 18.5 and 23.9 kg/m^2^ as the normal interval. Our study used the reference standards of China [2, 9, 10]. According to BMI groups, there were significant differences between the PA values of six parts. We found that the PA values of the six parts showed an increasing trend with the increase in BMI. With the exception for the 0–1 group, the PA of the six parts was inversely related to PG-SGA, which was statistically significant (*p* < 0.05). This finding may be related to the very small proportion of patients in the 0–1 group, which was only 1.77%.

According to the CSONSC recommends, PG-SGA ≥ 4 was used as the cut-off point for the diagnosis of malnutrition in clinical practice. Because of the relatively good data concentration and the best overall correlation with other parts, we use the PA-WB for the regression analysis. We used ROC curve to compare the diagnostic value of TR-WB and combined model in malnutrition. The AUC of both were greater than 0.5, which had a certain diagnostic value. The statistic Z was calculated according to the AUC and SE of the two ROC curves. *Z* = 2.577, *p* = 0.008 < 0.05. The difference of AUC was statistically significant(*p* < 0.05). It can be considered that the prediction model is better than PA-WB in judging malnutrition.

In summary, like BMI and PG-SGA, the PA values of six parts can also be used for nutritional assessment of patients with malignant tumors, which will greatly improve the efficiency of medical staff and save labor costs. However, it should be noted that the PA reference values of different body parts for judging malnutrition are different. The prediction model constructed by age, BMI, and PA-WB can be used as a useful tool for nutritional evaluation of tumor patients.

Our study focuses on the application value of PA values of six body parts for the diagnosis of malnutrition, whether nutritional intervention can significantly increase the PA value and whether increased PA can improve the clinical outcome. Further research is needed to confirm.

## Data Availability

Some or all data generated or used during the study are available from the corresponding author by request.
